# The phylogeny of 48 alleles, experimentally verified at 21 kb, and its application to clinical allele detection

**DOI:** 10.1186/s12967-019-1791-9

**Published:** 2019-02-11

**Authors:** Kshitij Srivastava, Kurt R. Wollenberg, Willy A. Flegel

**Affiliations:** 1 Laboratory Services Section, Department of Transfusion Medicine, NIH Clinical Center, National Institutes of Health, Bethesda, MD 20892 USA; 2 Bioinformatics and Computational Biosciences Branch, Office of Cyber Infrastructure and Computational Biology, National Institute of Allergy and Infectious Diseases, Bethesda, MD USA

**Keywords:** Allele prediction, Next generation sequencing, Phylogeny, Scianna, *ERMAP*

## Abstract

**Background:**

Sequence information generated from next generation sequencing is often computationally phased using haplotype-phasing algorithms. Utilizing experimentally derived allele or haplotype information improves this prediction, as routinely used in HLA typing. We recently established a large dataset of long *ERMAP* alleles, which code for protein variants in the Scianna blood group system. We propose the phylogeny of this set of 48 alleles and identify evolutionary steps to derive the observed alleles.

**Methods:**

The nucleotide sequence of > 21 kb each was used for all physically confirmed 48 *ERMAP* alleles that we previously published. Full-length sequences were aligned and variant sites were extracted manually. The Bayesian coalescent algorithm implemented in BEAST v1.8.3 was used to estimate a coalescent phylogeny for these variants and the allelic ancestral states at the internal nodes of the phylogeny.

**Results:**

The phylogenetic analysis allowed us to identify the evolutionary relationships among the 48 *ERMAP* alleles, predict 4243 potential ancestral alleles and calculate a posterior probability for each of these unobserved alleles. Some of them coincide with observed alleles that are extant in the population.

**Conclusions:**

Our proposed strategy places known alleles in a phylogenetic framework, allowing us to describe as-yet-undiscovered alleles. In this new approach, which relies heavily on the accuracy of the alleles used for the phylogenetic analysis, an expanded set of predicted alleles can be used to infer alleles when large genotype data are analyzed, as typically generated by high-throughput sequencing. The alleles identified by studies like ours may be utilized in designing of microarray technologies, imputing of genotypes and mapping of next generation sequencing data.

**Electronic supplementary material:**

The online version of this article (10.1186/s12967-019-1791-9) contains supplementary material, which is available to authorized users.

## Background

Exact matching for alleles improved survival following bone marrow transplantation [1] and reduced alloimmunization in chronically transfused patients [2–4]. Using computational algorithms, the large genotype datasets from next generation sequencing (NGS) can be phased into alleles or haplotypes [5, 6]. Using family relationships or applying experimentally confirmed allele information improves the inference accuracy, as routinely demonstrated in clinical HLA typing [7]. Blood group genes are less polymorphic than the highly variable, often shorter, *HLA* genes. Out of the 36 blood group systems and the genes encoding them, experimentally confirmed alleles are known for short genes only, such as *ICAM4* [8] and *ACKR1* [9]. For longer genes, such as *ABO* and *ERMAP* of more than 20 kb, and linked genes, such as *RHD* and *RHCE*, most haplotypes had only been computationally predicted [10–12].

The *ERMAP* gene, located on chromosome 1, encodes the glycoprotein carrying the antigens of the Scianna blood group system (SC; ISBT 013) in humans [13–15]. The single-pass transmembrane glycoprotein is likely involved in cell adhesion and recognized by immune cells [13, 16, 17]. The gene belongs to the butyrophilin (BTN) family which is a type 1 membrane protein of the immunoglobulin (Ig) superfamily [18]. The butyrophilin and butyrophilin-like proteins have recently been studied as potentially important immune regulators [19, 20].

We have previously assessed the nucleotide variations in the *ERMAP* gene and unambiguously identified 48 alleles at 21,406 nucleotides each in 50 unrelated individuals from 5 different populations [21]. We propose using the phylogeny of this set of 48 alleles and identifying evolutionary steps to derive the observed alleles [22]. We predicted unobserved alleles at every internal node and their posterior probabilities. These inferred alleles, represented by sequences identified in the nodes, are possible candidates for alleles segregating in the population. Our new approach proposes a method of utilizing not-yet-observed alleles, predicted by phylogeny, for phasing patient genotypes in clinical diagnosis and therapy.

## Methods

The sequence information for 48 *ERMAP* alleles was retrieved from GenBank (KX265189–KX265236) [21]. The phylogenetic tree was rooted using the chimpanzee *ERMAP* sequence as outgroup (GenBank number NC_006468.4; range 42,268,258 to 42,295,767). Full-length sequences were aligned using the MAFFT version 7 program [23]. All of the 72 variable sites were extracted manually from the 48 *ERMAP* alleles [21]. The Bayesian coalescent algorithm implemented in BEAST v1.8.3 [24] was used to estimate a coalescent phylogeny for these variants and the allelic ancestral states at the internal nodes of the phylogeny. All analysis was done using default parameters. Internal node is a theoretical representation of a common ancestor between sampled alleles and are often extant in population level studies [25]. If more than one mutational or recombinational step is required to join some nodes, predicted alleles are incorporated to complete the tree [26].

We executed 4 independent runs of the program, each using the Tamura-Nei substitution model [27], a lognormal relaxed clock model [28], and a constant-size coalescent model [29]. After 40 million generations the parameter estimates were examined and determined to have converged for each run. The allelic ancestral states at each node and their posterior probabilities were extracted manually from the maximum clade compatibility tree estimated from 9001 Markov chain Monte Carlo samples generated by the BEAST software. For the ancestral allele reconstructions, we generated a set of all possible ancestors for each node and selected the predicted allele with the highest posterior probability.

## Results

**Fig. 1:**
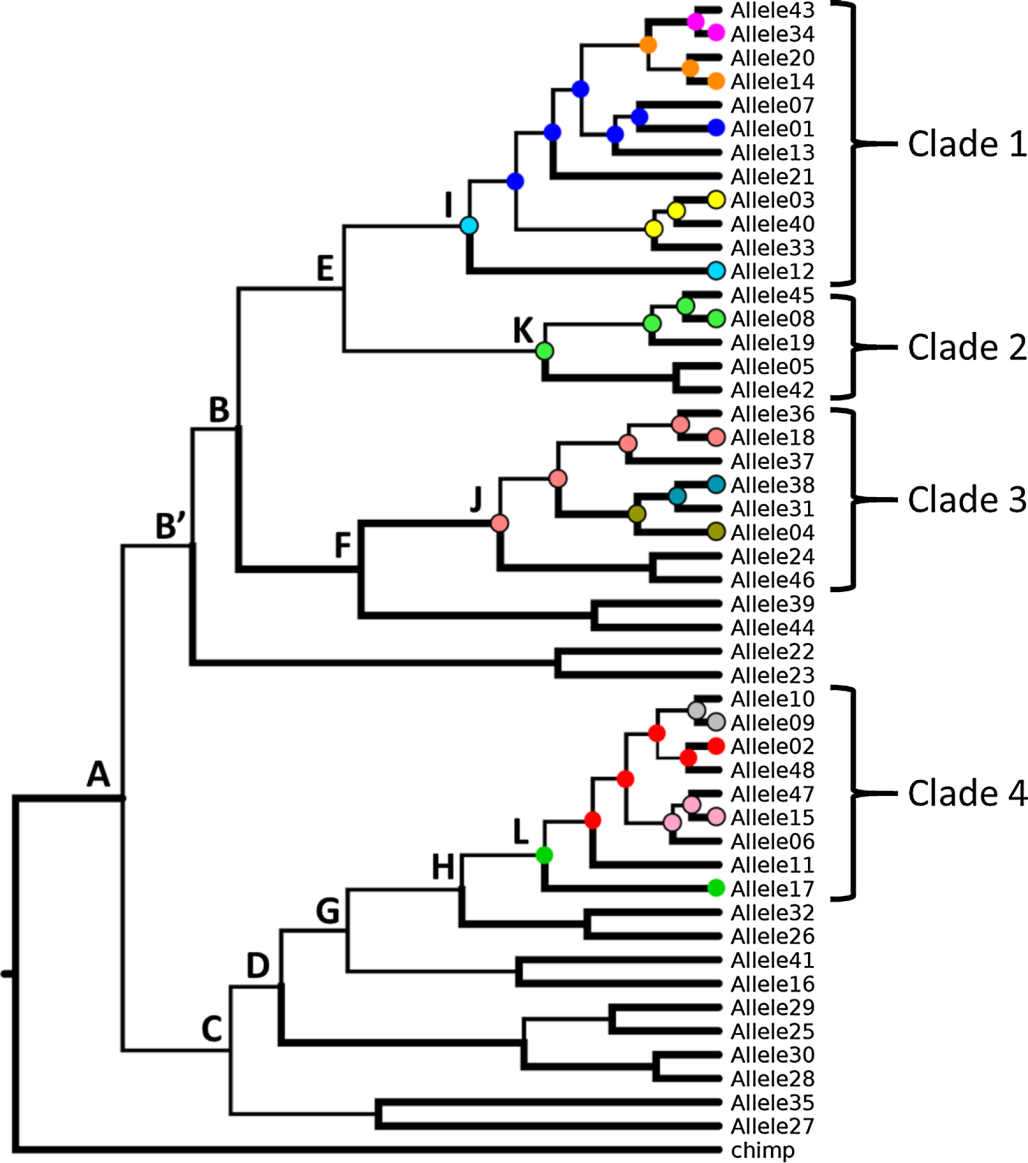
Phylogenetic tree of 48 *ERMAP* alleles. The phylogeny of the 48 known *ERMAP* alleles was determined based on a standard Bayesian phylogenetic analysis. Branch width indicates posterior probability support (thick is ≥ 0.95 and thin is < 0.95). The colored circles represent sampled alleles that are also predicted ancestral alleles with the highest posterior probability. The 13 nodes are labelled A to L. Nodes B and B′ share the same allele with the greatest, but different, posterior probabilities (see Table 1)

A Bayesian phylogeny of 48 previously published *ERMAP* alleles was calculated (Fig. 1). Based on this phylogenetic tree, we predicted alleles, many of which may be extant in the population, particularly those of greater posterior probability. Our approach applied standard methods of phylogenetic inference, ancestral character reconstruction and aimed to enrich the repertoire for a focal genomic region, of specific clinical interest.

### Phylogeny

The Bayesian phylogenetic analysis of the 48 *ERMAP* alleles identified 13 nodes (Fig. 1, nodes A to L) and 4 clades (Fig. 1, clades 1 to 4). The clades comprised clusters of 5 to 12 alleles. Alleles were equally distributed between African American and Caucasian populations (Additional file 1: Fig. S1). For each clade, one observed allele was identified as the ancestral allele and had a posterior probability of more than 0.60 (nodes I to L). The remaining 9 internal nodes had 8 predicted alleles as the most probable ancestors with the highest posterior probabilities ranging from 0.235 to 0.792 (nodes A to H; Table 1). Thus, the phylogenetic tree comprised 4 confirmed alleles and 8 predicted alleles (Table 1). The most likely ancestral allele (node A; posterior probability = 0.235) for all 48 *ERMAP* alleles had only 4 nucleotide differences relative to our reference sequence (GenBank accession KX265235).

**Table 1:** Predicted alleles at internal nodes of the *ERMAP* phylogeny

Node	Allele^a^	Sequence^b^	Posterior probability	Status	GenBank number
Reference	Allele1	ATTGGCACCAGGCCGCCGCCCTGCTTAAGCCCTGGCGTGGTACTCGTCACGGTCCGCCGGGGCCGGATTAAA	1	Observed	KX265235
A	SPA18	------G--------------G--------T-------------T---------------------------	0.235	Predicted	na
B	SPA03	------G--------------G--------T-----------------------------------------	0.792^c^	Predicted	na
C	SPA06	------G----------A---G--------T-----------T-T---------------------------	0.444	Predicted	na
B′	SPA03	------G--------------G--------T-----------------------------------------	0.516^c^	Predicted	na
D	SPA09	------G----------A---G--------T---A-A-----T-T---------------------------	0.608	Predicted	na
E	SPA04	------G--------------G--------------------------------------------------	0.747	Predicted	na
F	SPA07	------G-------------TG--G--G--T-----------------------------------------	0.626	Predicted	na
G	SPA10	-C----G----------A---G--------T---A-A-----T-T---------------------------	0.594	Predicted	na
H	SPA13	-C----G----------A---G----T---T---A-A-----T-TC--------------------------	0.492	Predicted	na
I	Allele12	---------------------G--------------------------------------------------	0.621	Observed	KX265198
J	Allele18	G-----G---A--------TTG--G--G--T-----------------------------------------	0.674	Observed	KX265204
K	Allele08	------G--------------G-------------T------------------------------------	0.888	Observed	KX265194
L	Allele17	-C----G----------A---G----T---T---A-A---C-T-TC--------------------------	0.634	Observed	KX265203

*na* not applicable

^a^Alleles 1, 8, 12, 17, and 18 are experimentally confirmed alleles as published previously [21]. SPA03—SPA18 are predicted alleles (see Additional file 1: Table S1)

^b^The nucleotides at the 72 SNP positions with variations are shown in 5′ to 3′ orientation (Table S2 in Srivastava et al. [21])

^c^The posterior probabilities differ for SPA03 depending on its position in the phylogenetic tree (see Fig. 1)

### Ancestral allele prediction

From the phylogenetic tree, we extracted all possible ancestral alleles at each internal node (nodes A to L). A total of 4243 unique predicted alleles were computed and sorted according to their calculated posterior probability of being the true ancestor (Additional file 2: Excel file S1). Even though the posterior probabilities of the inferred ancestral alleles were often below the threshold for statistical significance (0.95), the posterior probabilities of the next most likely predicted alleles dropped off dramatically. The exceptions to this were at Node A (best posterior probability = 0.23, next best = 0.19), Node B′ (0.52 vs. 0.29), and Node I (0.62 vs. 0.34). In all other cases the posterior probabilities of the secondary inferred ancestral allele were less than half the greatest values.

## Discussion

A phylogenetic analysis was applied to a set of 48 physically confirmed *ERMAP* alleles covering 5 populations worldwide [21]. We predicted 4243 unobserved alleles and their distinct posterior probabilities. The relatively small number of predicted alleles contrasted to the vastly larger number of theoretically possible alleles. The predicted alleles have a stronger support for being correct and extant in the population because they are more likely ancestral to the observed alleles. We propose the concept of detecting unobserved, likely novel, alleles based on the phylogeny of verified alleles.

**Fig. 2:**
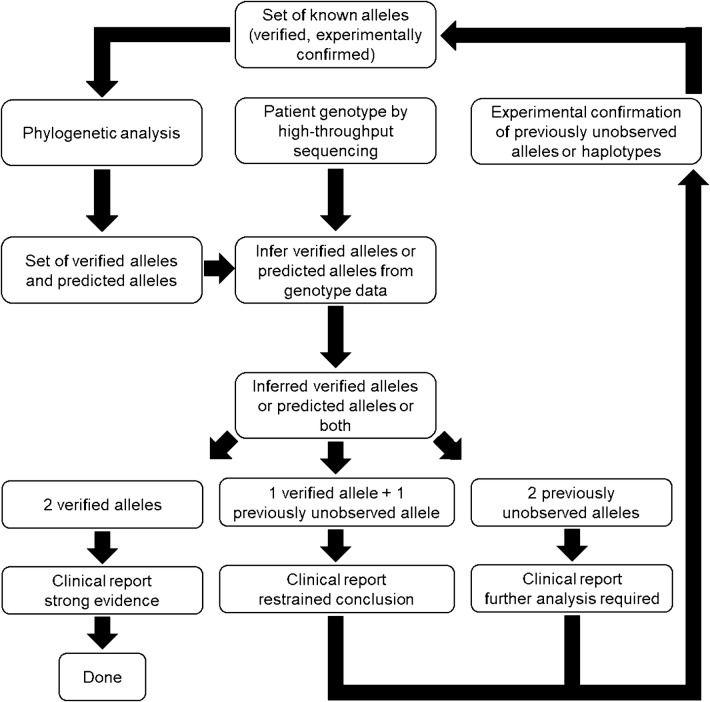
Algorithm to analyze genotypes and determine alleles using phylogeny data. Patient or blood donor genotype information for a particular gene is phased into alleles or haplotypes using statistical algorithms for clinical decisions. We propose a novel approach where the confidence for the inferred allele is based on verified, experimentally confirmed alleles and predicted alleles (see Fig. 1). The posterior probability of the predicted alleles is determined by a Bayesian phylogenetic analysis. Whenever a new allele is observed and experimentally confirmed, the phylogenetic analysis is in turn used to predict an updated set of alleles and their posterior probabilities. While this loop process continues, previously unobserved alleles will be encountered less frequently, as the set of confirmed allele increases

Previous computationally driven algorithms to phase NGS data such as read-backed phasing [30] and haplotype improver [31] remain very useful for phasing haplotypes and alleles in a population sample but may fail when applied to a single observation in an individual patient. Our approach utilizes predicted alleles and their posterior probabilities along with the verified alleles as templates for phasing the genotypes detected in high-throughput sequencing (Fig. 2), complementing the computationally driven algorithms. This approach increases the effective number of templates available for phasing and thus the accuracy of phased haplotypes and alleles. When a previously unobserved allele matches one of the predicted alleles, its posterior probability allows to quantify the reliability of the estimate for clinical decisions, such as in transfusion and transplantation settings, in a patient who bears a new allele. While the validation of the predicted alleles by applying our protocol was not performed in this study, the novel approach illustrates the potential use of phylogenetic data in a clinical diagnostic setting.

Our approach relies heavily on the accuracy of the alleles used for the phylogenetic analysis. Hence, reference sequences from online databases such as GenBank should be avoided as long as the information is not sufficiently replicated or independently verified [32]. The prevalence of the 48 alleles derived from 5 populations worldwide, but may still bias the imputation of novel alleles. Hence, addition of other alleles that are considered accurate, although computationally rather than physically derived, will strengthen the phylogenetic analysis and contribute to phasing of haplotypes and alleles, such as computed from the 1000 Genomes project [33] and similar online databases.

In our previously published set of long range *ERMAP* alleles with 72 single nucleotide polymorphisms (SNPs), the number of theoretically possible alleles was 2^72^ [21]. However, it is known that the majority of the haplotype diversity is constituted by only few common haplotypes, which is constant in a given population [34]. Our algorithm restricts the possible *ERMAP* alleles from 2^72^ to 4243 only, some associated with greater probability of being correct, but all as potential precursors of the experimentally verified extant alleles. With only 72 variable nucleotide positions in our set of 48 *ERMAP* alleles [21], the vast majority of positions remained uninformative (21,334 of 21,406 nucleotides: 99.66%).

Our observation contrasts with the 2353 SNPs, including 66 out of our 72 SNPs, reported for this DNA stretch covering the *ERMAP* gene [35], most of them being rare and often not validated to the extent needed for clinical decision making. Increasing the sample size will result in the confirmation of many or most of the previously reported 2353 SNPs and also the identification of novel SNPs in this DNA stretch. However, many of these SNPs will be specific for a small number of individuals resulting in a small global allele frequency.

While initially disregarding recombination as a major contributor, the subsequent analysis of the *ERMAP* sequences using the ClonalFrameML software [36] was also unable to detect any recombination event among the 48 confirmed alleles. This observation could be explained by the small sample size, which will resolve with the accumulation of more data. Our observation may, however, be an actual feature of *ERMAP* alleles in the population, because it is similar to the *ABO* gene, for which the detected recombinant alleles are also of low frequency [37]. As *ERMAP* alleles caused by recombination will eventually be found, they can be incorporated in the set of alleles used to compute the phylogenetic analysis.

## Summary

By applying a Bayesian phylogenetic approach to 48 alleles, more than 21 kb long and all experimentally verified, we predicted a large set of not-yet-observed alleles of the *ERMAP* blood group gene. We propose a strategy of using these predicted alleles and their associated probabilities of correctness in clinical diagnostics such as designing of microarray technologies, imputing of genotypes and mapping of NGS data.

## Additional files

**Additional file 1.** **Table S1.** Predicted *ERMAP* alleles with posterior probability of greater than 0.10. **Figure S2.** Distribution of alleles in 5 ethnic groups. The number of alleles observed in 50 individuals, as previously reported in Srivastava et al. (Table S2) [21], are shown for the clades in the phylogenetic tree (see Fig. 1).

**Additional file 2.** **Excel file S1.** List of 4243 predicted alleles of *ERMAP* gene.
