# Supplementary material for: The phylogeny of 48 alleles, experimentally verified at 21 kb, and its application to clinical allele detection
Source: J Transl Med. 2019 Feb 11;17:43. doi: 10.1186/s12967-019-1791-9 (PMC6371619; doi:10.1186/s12967-019-1791-9)
Supplement: Supplementary file 1 — Additional file 1. Table S1. Predicted ERMAP alleles with posterior probability of greater than 0.10. Figure S2. Distribution of alleles in 5 ethnic groups. The number of alleles observed in 50 individuals, as previously reported in Srivastava et al. (Table S2) [21], are shown for the clades in the phylogenetic tree (see Fig. 1). [file 12967_2019_1791_MOESM1_ESM.pdf]

Table S1. Predicted *ERMAP* alleles with posterior probability of > 0.10

Allele number	Sequence[a]	Posterior probability	Status	GenBank number
Reference	ATTGGCACCAGGCCGCCGCCCTGCTTAAGCCCTGGCGTGGTACTCGTCACGGTCCGCCGGGGCCGGATTAAA	1	Observed	KX265235
SPA01	------G-------------TG--G--G--T---------C-------------------------------	0.849	Predicted	na
SPA02	------G----------A---G--------T-C-A-A-A--GT-T---------------------------	0.841	Predicted	na
SPA03	------G--------------G--------T---	0.792	Predicted	na
SPA04	------G--------------G--	0.747	Predicted	na
SPA05	------G----------A---G--------T-C-ACA-A--GT-T---T-C--------------------G	0.671	Predicted	na
SPA06	------G----------A---G--------T-----------T-T---------------------------	0.669	Predicted	na
SPA07	------G-------------TG--G--G--T---	0.626	Predicted	na
SPA08	G-----G---A--------TTG--G-----T--------------------A-------A------------	0.615	Predicted	na
SPA09	------G----------A---G--------T---A-A-----T-T---------------------------	0.608	Predicted	na
SPA10	-C----G----------A---G--------T---A-A-----T-T---------------------------	0.594	Predicted	na
SPA11	------G--------------G--------T--------A-GT-----------------------------	0.532	Predicted	na
SPA12	-C----G--------------G-------------T-------CT----G----------------------	0.521	Predicted	na
SPA13	-C----G----------A---G----T---T---A-A-----T-TC--------------------------	0.492	Predicted	na
SPA14	-CC---G-----G----AT--G----T---T---A-A-----T-TC--------------------------	0.451	Predicted	na
SPA15	-C----G----------A---G----T---T---A-A---C-T-TC---G----------------------	0.416	Predicted	na
SPA16	-C----G----------A---G--------T---A-A-------T---------------------------	0.399	Predicted	na
SPA17	------G--------------G--------T-----------T-----------------------------	0.294	Predicted	na
SPA18	------G--------------G--------T-------------T---------------------------	0.235	Predicted	na
SPA19	------G---A--------TTG--G-----T--------------------A-------A------------	0.226	Predicted	na
SPA20	------G-------------TG--G-----T---	0.148	Predicted	na
SPA21	------G----------A---G--------T-C-A-A----GT-T---T-C--------------------G	0.140	Predicted	na
SPA22	G-----G---A--------TTG--G--G--T-----------------T-C-------A------------G	0.117	Predicted	na
SPA23	------G---A--------TTG--G--G--T---	0.110	Predicted	na
SPA24	------G--------------G--------T-----------T-T---------------------------	0.110	Predicted	na
SPA25	------G----------A---G--------T-C-A-A----GT-T---------------------------	0.107	Predicted	na

na ━ not applicable

a The nucleotides at the 72 SNP positions with variations are shown in 5‘ to 3’orientation (Table S2 in Srivastava et al. [21]).

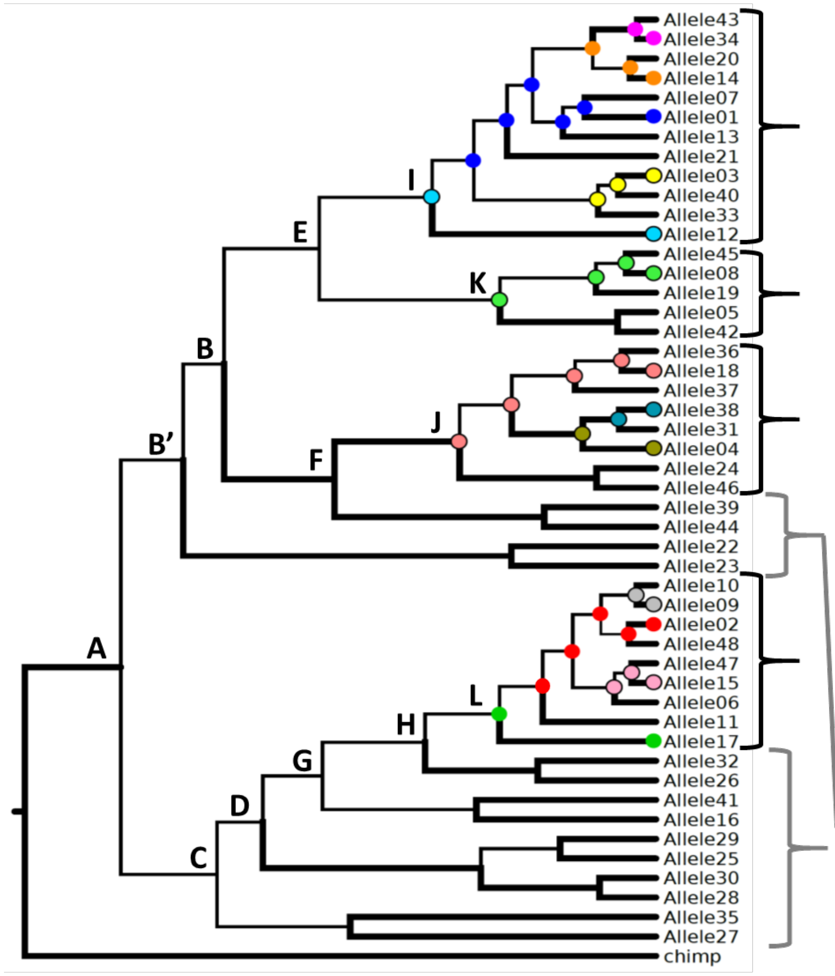

	Observations (n, %)				
	African American	Caucasian	Thai	Hispanic	Asian
	14<br>35%	7<br>35%	5<br>25%	2<br>20%	2<br>20%
	3<br>7.5%	2<br>10%	2<br>10%	2<br>20%	2<br>20%
	4<br>10%	4<br>20%	0<br>0%	3<br>30%	2<br>20%
	9<br>22.5%	6<br>30%	11<br>55%	2<br>20%	3<br>30%
	10<br>25%	1<br>5%	2<br>20%	1<br>10%	1<br>10%
Total	40<br>100%	20<br>100%	20<br>100%	10<br>100%	10<br>100%

Figure S1
